# Metal-Based Nanomaterials: Fabrications, Optical Properties, and Ultrafast Photonics

**DOI:** 10.3390/nano15030186

**Published:** 2025-01-24

**Authors:** Bo Fu, Vittorio Scardaci

**Affiliations:** 1 Hangzhou International Innovation Institute, Beihang University, Hangzhou 311115, China; 2 Key Laboratory of Precision Opto-Mechatronics Technology of Education Ministry, School of Instrumentation and Optoelectronic Engineering, Beihang University, Beijing 100191, China; 3 Dipartimento di Scienze Chimiche, Università degli Studi di Catania, Viale A. Doria 6, 95125 Catania, Italy

Metals are known for conductivity and luster due to the abundance of free electrons. Owing to these electrons, metals can support surface plasmon resonance (SPR) which increases their sensitivity to light intensity and the refractive indices of the materials. Nonlinear phenomena such as saturable absorption arise subsequently, leading to the utilization of metal materials in lasers, spectroscopy, and metasurfaces [1,2,3]. The properties of metals change significantly when they are engineered into nanomaterials. SPR in metal nanoparticles is often referred to as localized SPR, where the plasmon oscillations are confined to the surfaces of nanoparticles rather than propagating along the interface between the metal and the dielectric. This effect endows metal nanomaterials with enhanced optical sensitivity, as well as a tunability that is dependent on the shape of the nanoparticle [4]. Meanwhile, metallic compound nanomaterials such as transition-metal dichalcogenide are also widely used in ultrafast lasers, although their optical properties are primarily governed by the bandgap since they exhibit a weaker SPR due to their lack of free electrons [5]. In addition, metals and other materials can enhance each other in metal-based composite nanomaterials, providing opportunities to discover materials with better performance [6]. Four research articles and one review are presented in this Special Issue on “Metal-Based Nanomaterials: Fabrications, Optical Properties, and Ultrafast Photonics”. We aim to highlight the influence of metallic elements on the optical properties of materials, thereby supporting advancements in the fields of biology, medicine, and sensing.

Janus nanoparticles, named after the two-faced Roman god, are a class of asymmetric nanoparticles characterized by different properties on their opposite surfaces [7]. In metal-based Janus nanoparticles, metals such as gold and platinum have been deposited onto polymers or silica in order to provide asymmetric SPR, making them suitable for left-handed materials (LHMs) [8]. LHMs are characterized by their simultaneous negative permittivity and permeability, which lead to special electromagnetic properties such as negative refraction [9]. Early LHMs were artificial periodic structures, while the newly discovered disordered structures, including Janus nanoparticles immersed in positive-permittivity hosts have shown better robustness and cost efficiency. Recently, Bărar et al. extended the classical effective medium methods to describe the electromagnetic properties of Janus nanoparticle-based LHMs [10]. The authors adapted the Maxwell–Garnett and Bruggeman mixing laws for better simulations of LHMs, and analyzed the unique fundamental permittivity, conductivity, and dispersion properties of LHMs. This work provides a quantitative framework for analyzing Janus nanoparticle-based LHMs, which helps researchers to create new ultra-compact lenses, electromagnetic cloaks, and subwavelength imaging systems.

Metal-based nanomaterials have been known for their usage in pulsed lasers, and now their combination with 2D materials may offer better performance. In passively mode-locked or Q-switched lasers, saturable absorbers play a crucial role in modulating the laser pulses by manifesting a lower absorption of light with a higher intensity. Various nanomaterials, such as carbon nanotubes, graphene, metal nanoparticles, transition metal dichalcogenides, and heterostructure materials, have been explored as saturable absorbers for their nonlinear absorption [11]. Among them, Ag nanoparticles stand out for their large third-order nonlinearity and strong SPR [12]. MXenes, a family of two-dimensional transition metal carbides/nitrides, exhibit high electrical conductivity and a tunable bandgap [13]. Zhao et al. discovered that when these two materials were combined, the resulting Ag/MXene composite obtained better nonlinear absorption. They demonstrated a Tm:Ho co-doped fiber laser operating at 2 μm with Ag/MXene as the saturable absorber [14]. The interaction between Ag and MXene extends the lifetime of electrons excited by laser beam in MXene, thereby strengthening the bleaching effect and improving the modulation depth and saturation intensity of the composite. This finding suggests that with due synthesis, nanocomposites are able to achieve better ultrafast properties than a single material, and contribute to high-performance pulsed fiber lasers for optical and medical applications.

In the realm of advanced optical materials, the introduction of post-transition metal cations including Pb^2+^, Sn^2+^, Ba^2+^, and Sr^2+^ into crystals is an effective way to influence optical properties [15]. These cations can provide strong spin–orbit coupling (SOC) effects due to their high atomic numbers and relativistic effects. This strong SOC results in band splitting, where degenerate energy levels split into multiple sublevels with different spin orientations [16]. SOC plays a crucial role in modifying electronic structures, and is related most notably to quantum computation [17]. Noticing that the significance of SOC effects had been underrated in research on optical materials, Leng et al. provided a comprehensive first-principles investigation into the X_2_PO_4_I (X = Pb, Sn, Ba, and Sr) series. In their work, the SOC effects on the optical properties were analyzed with density functional theory [18]. They revealed that SOC induces significant band splitting, reduces band gaps, and enhances the stereochemical activity of lone-pair electrons, leading to changes in electronic structure, refractive indices, and birefringence. This research stresses the important roles that metal elements play in optical properties, and offers valuable insights for designing novel optical materials with tailored functionalities.

Metal-based nanoparticles can also enhance the fluorescence-based technology in chemistry, biology, and sensing. Random lasers are a unique class of lasers that rely on scattering media, rather than traditional optical cavities, to achieve coherent light emission. Metal-based nanoparticles such as TiO_2_ and ZnO are often used in random lasers as scatterers due to their high refractive index, which enhances light scattering and promotes feedback within the gain medium [19,20]. Meanwhile, in the biological field, fluorescence and laser technologies are frequently used for imaging and diagnostics. They often utilize the scattering properties of biological tissues or added nanoparticles to enhance signal detection [21]. In order to combine the concepts of random and biological lasers, Bonnefond et al. demonstrated a fluorescence amplification technique using TiO_2_ nanoparticles as nanoscatterers and fluorescein-5-isothiocyanate as the fluorescent dye in a biological pH environment [22]. The study shows a fluorescence amplification of up to a factor of 40, as well as significant spectral narrowing and reduced fluorescence pulse duration, indicating the contribution of nanoscatterers. Furthermore, TiO_2_ is generally known as economical, environmentally friendly, and relatively safe for the human body, offering a promising way to enhance fluorescence signals in biological applications.

Finally, metal-based nanomaterials have the potential to facilitate polarization holography. Holography is a technique that records and reconstructs three-dimensional light fields, wherein polarization holography put a special focus on the recording of polarization [23]. This requires materials with appropriate photoinduced birefringence, which increases the diffraction efficiency of the recorded polarization diffraction gratings. Azopolymer materials have been preferred as the main type of recording mediums owing to their trans–cis–trans photoisomerization [24]. Later, researchers found that the incorporation of certain metallic or non-metallic nanoparticles into these azopolymer matrices can result in a significant enhancement in their holographic performance. Among the metal-based nanoparticles, gold, silver, ZnO, and TiO_2_ have been used in several studies. This enhancement is attributed to three possible mechanisms: nanoparticles may increase the free volume, the scattering, and in the case of metal nanoparticles, the SPR. Nazarova et al. provided a comprehensive review of this field, and listed key parameters such as birefringence, diffraction efficiency, and surface relief height in recent works as a characterization of performance [25]. These findings show that metal-based nanocomposites are promising in optical applications for the unique properties induced by the combination of different materials.

In summary, this Special Issue introduced special metal-based nanocomposites including Janus nanoparticles and Ag/MXene, the optical impact of post-transition metal elements in compounds, as well as applications in fluorescence-based technology and polarization holography. We expect that this Special Issue will encourage researchers in optics and materials science to explore more aspects of metal-based nanomaterials in terms of their fabrication and applications, so that their extraordinary optical properties can be further exploited.
